# Comments: Absolute cardiovascular risk assessment for guiding antihypertensive prescribing in Australian primary care patients with hypertension

**DOI:** 10.1038/s41371-022-00719-4

**Published:** 2022-07-06

**Authors:** Woldesellassie M. Bezabhe, Gregory M. Peterson

**Affiliations:** School of Pharmacy and Pharmacology, University of Tasmania, Private Bag 26, Hobart, TAS 7001 Australia

**Keywords:** Risk factors, Preventive medicine

Cardiovascular disease (CVD) is a leading cause of death in Australia [1]. Hypertension is one of the most important modifiable CVD risk factors, and treatment with antihypertensive agents significantly reduces morbidity and mortality. Australian guidelines recommend antihypertensive drug prescribing guided by the absolute CVD risk score instead of a single risk factor, blood pressure [2]. Based on this score, patients are categorized as high-risk (>15% likelihood of CVD event over five years), moderate-risk (10–15%), or low-risk (<10%). The absolute CVD score recognizes the cumulative and synergistic effect of multiple CVD risk factors [2] and has overall benefit in guiding preventive measures to patients at greater risk [3]. Antihypertensive agents are recommended for patients with a high absolute CVD risk score. However, their use in moderate-risk patients depends on blood pressure values, family history of premature CVD, and ethnic background. Some groups of patients (with diabetes and aged more than 60 years, diabetes and microalbuminuria, moderate to severe chronic kidney disease, a previous diagnosis of familial hypercholesterolemia, systolic blood pressure ≥ 180 mmHg or diastolic blood pressure ≥ 110 mmHg, or serum total cholesterol > 7.5 mmol/L) are automatically classified as clinically high-risk regardless of their absolute CVD risk score and are recommended to receive antihypertensive therapy [2].

Collaborative efforts have been implemented to reduce the burden of CVD in Australia. These include governmental, non-governmental and professional bodies advocating the use of absolute CVD scores. The absolute CVD score guidelines were produced by the National Vascular Disease Prevention Alliance (NVDPA), an alliance of four Australian non-government organizations (National Stroke Foundation, Diabetes Australia, Kidney Health Australia, and the Heart Foundation) [2]. An absolute CVD risk score is dynamic, and the Australian guidelines recommend reassessing it at least every two years [2]. The Australian government funds programs to lessen the burden of CVD. For instance, the Practice Incentives Program Quality Improvement (PIP QI) and Heart Health Checks are subsidized through the Medicare Benefits Schedule to encourage CVD risk factors to be identified and CVD risk scores calculated, recorded and managed [4].

NPS MedicineWise has encouraged absolute CVD risk assessment through education and quality improvement programs in general practice. Additionally, as of March 2020, it had enrolled 732 general practices across Australia and has been collecting de-identified data regularly from these practices for its dataset, MedicineInsight [5]. Analysis of this dataset has been used to evaluate and promote the quality use of medicines. The analysis of this national dataset by Roseleur et al. [6]. provided an insight into what has been achieved in assessing the absolute CVD score and using it to guide antihypertensive prescribing in Australian primary care patients. It included 571,492 patients aged between 45 and 74 years and found that antihypertensive prescribing was lower in patients with high absolute CVD risk score (57.4% 65 (95% CI: 55.4–59.4)) compared with those at low-risk (63.3% (95% CI: 61.9–64.8)) or moderate-risk (61.8% (95% CI: 60.2–63.4)) or at high-risk clinically [64.1% (95% CI: 61.9–66.3)) [6]. Also, almost half of patients with hypertension had insufficient recorded data to calculate their absolute CVD risk scores. These findings suggest nonadherence to hypertension guidelines recommendations and might indicate not monitoring and recording CVD risk factors, not calculating absolute CVD risk score and not using it to guide antihypertensive prescribing. These findings agree with a previous analysis of MedicineInsight data by Raffoul et al. [7]., which found only 17% of patients aged 45–74 years regularly attending general practice had all the relevant risk factors recorded to enable absolute CVD risk assessment.

The Roseleur study [6] has several limitations, such as the inability to determine the baseline CVD risk before initiation of antihypertensives; the most recent blood pressure and cholesterol measures were used to calculate CVD risk. The study could have been more informative if it had shown the trends in CVD score recording, antihypertensive prescribing, and absolute CVD risk reclassification. In a follow-up study [8] using MedicineInsight data, we longitudinally reassessed the stroke risk of patients with atrial fibrillation. We found that the CHA_2_DS_2_-VA score increased by 1.10 (95% CI, 1.01–1.20), 1.63 (95% CI, 1.53–1.72), and 1.32 (95% CI, 1.26–1.38) points for the baseline low, moderate and high stroke risk categories, respectively, during 9.4 ± 1.0 years of follow-up [8]. Approximately one-third of patients reclassified as being at high risk of stroke were not prescribed oral anticoagulant therapy. When prescribed, there was a median of 2 years delay in oral anticoagulant initiation following reclassification to high risk from baseline low/moderate risk [8].

The Roseleur study [6] identified the limitation of not tracking patients when visiting practices not enrolled in MedicineInsight and included regular patients who visited the practices at least three times in two years, between 2016 and 2018. However, it did not determine whether more frequent visits were associated with better absolute CVD risk monitoring and antihypertensive prescribing [9]. Khanam et al. [10], using the same dataset, demonstrated that patients with chronic kidney disease with higher relational continuity of care and more general practitioner visits were more likely to achieve blood pressure targets. At the same time, this was less likely when the target blood pressure was lowered by concomitant diabetes or cardiovascular disease.

Roseleur et al. [6] have identified clear gaps between clinical guideline recommendations and practice in assessing the absolute CVD risk score and using it to guide antihypertensive prescribing in Australia. Alternative strategies appear to be needed. Perhaps directly educating the public to know their absolute CVD risk score might help? Future studies could determine whether more frequent visits to a general practitioner are associated with better assessment and recording of absolute CVD scores, antihypertensive prescribing, and blood pressure control. It would also be of interest to evaluate the changes in absolute CVD score over time and the delay in antihypertensive prescribing in patients with hypertension reclassified as high absolute CVD risk.
